# Editorial: Advances in antimicrobial therapy and combating resistance

**DOI:** 10.3389/fphar.2023.1170289

**Published:** 2023-03-16

**Authors:** Islam M. Ghazi, Wasim S. El Nekidy

**Affiliations:** ^1^ Long Island University-Brooklyn, Brooklyn, NY, United States; ^2^ Cleveland Clinic Abu Dhabi, Abu Dhabi, Abu Dhabi, United Arab Emirates

**Keywords:** polymyxin, bacterial resistance, stewardship, tuberclosis, pharmakocinetics

The progression of microbial resistance and consequent failure to eradicate infections, clinically opposes one of the major human strides in medicine, which is the discovery of antimicrobials. The rapidly evolving microbial defense systems are paralleled by relentless efforts of scientists and clinicians to prolong the era of effective antimicrobials. Design of novel molecular entities, rational clinical use, optimization of pharmacokinetic exposure, and adherence to stewardship principles are examples of strategies to halt the development of microbial resistance. In this Research Topic we called upon researcher to publish their endeavors in this arena.

The rediscovery of older effective antibiotics has recently gained a momentum probably due to the dwindling number of new antibiotics joining the therapeutic armamentarium and diminishing industry investment. Jia et al. retrospectively studied the salvage treatment of a group of Chinese children infected with carbapenem resistant Gram-negative bacteria with polymyxin B. The antibiotic (mostly used in combination with other regimens) demonstrated efficacy in 52.7% of the cases. However, this efficacy was accompanied with 27.3% acute kidney injury (AKI). The authors also highlighted that appropriate dose and therapy duration may improve outcomes. Furthermore, the loading dose did not improve efficacy and avoiding electrolyte imbalances may mitigate toxicity.

In a prospective clinical pharmacokinetic study investigating polymyxin B optimal exposure, Zhewei Zu et al., 2022, reported that *f*AUC_ss,24h_/MIC of ≥22.8 was associated with eradication of blood stream infections caused by carbapenem resistant *Klebsiella pneumoniae* (CRKP) with MIC≤ 1 mcg/mL. A Monte Carlo simulation revealed that a dose of 1.25 mg/kg every 12 h was efficacious against CRKP with such MIC. Polymyxin B—consistent with *in vitro* studies-demonstrated rapid concentration-dependent bactericidal effect achieving microbiological clearance around day 3 of therapy. The study also emphasized that the pharmacokinetics of the drug in patients with blood stream infections are quite distinctive from these in healthy subjects which could be related to the pathologic changes (Cl; 0.028 ± 0.007 L/kg/h and0.026 ± 0.004 L/kg/h—VD; 0.490 ± 0.142 L/kg and 0.204 ± 0.026 L/kg—T_1/2_; 12.5 ± 3.11 vs 5.55 ± 0.942 h, respectively). Finally, the authors attributed the incidence of 55.6% AKI at day 7 of therapy, to the high daily dose and cumulative exposure. Furthermore, they observed a 70% incidence of neurotoxicity and an overlap between exposures responsible for efficacy and emergence of adverse events.

A summary of studies discussing the need for dose adjustment in renally impaired patients, Nie et al., argued that polymyxin B is non-renally excreted as evidenced by urine recovery of 0.04%–0.86% of the dose in unchanged form. A case report of renal-impaired patients showed that the drug had comparable half-life of11.5 h similar to those with normal renal function. In a population PK study, there was a lack of correlation between polymyxin B and creatinine clearance. Additionally, in another PK study, the authors found comparable total polymyxin B exposures in both intact and impaired renal function patients who received administered comparable doses. Furthermore, another two studies illustrated no significant difference in incidence of adverse events, microbiological cure or 30-day mortality. On the other hand, several studies based on Monte Carlo simulations, demonstrated that creatinine clearance is a significant covariate in polymyxin B clearance, and the dose should be adjusted based on renal function. The authors of this opinion paper concluded that the evidence is insufficient to reach consensus recommendation.

Continuing with polymyxins, Xu-ben Yu et al., 2022, studied the pharmacokinetics of polymyxin E (colistin sulfate, administered as active form) in critically ill patients. The authors found that the drug clearance is similar to polymyxin B and dependent on creatinine clearance. Of the patients infected with carbapenem resistant organisms, 60% achieved clinical cure with analogous to polymyxin B. The study showed that colistin sulfate achieved comparable clinical cure rate similar to polymyxin B despite of the use of significantly lower doses which could be attributed to lower pharmacokinetic target based on higher potency and fraction of unbound drug. The above studies provided insights into the clinical use of polymyxins with respect to optimizing dosing and minimizing untoward effects which becomes particularly beneficial when treating carbapenem resistant bacteria in critically ill patients with multi-drug resistant organisms.

Tackling another Research Topic, the emergence of multi drug resistant *Mycobacterium tuberculosis* (MDR-TB), Azimi et al., conducted a systematic review and meta-analysis of the literature pertaining to the prevalence of TB resistance to linezolid (LNZ). The meta-analysis calculated prevalence (4.2%) of LNZ resistance among all MDR-TB isolate peaking in Spain (22%) and troughing (0.2%) in United States of America LNZ resistance to LND develops as a consequence of several genetic mutations leading to changes in the target binding site (23S rRNA). Combination of LNZ with other anti-tubercular medications can produce synergism, shorten treatment duration, and obliterate latent infections.

Since irrational prolonged antibiotic administration could promote the emergence of resistance, He et al., investigated the risk factors associated with prolonged antibiotic use in pediatrics with bacterial meningitis. The study revealed that even with use of guidelines recommended regimens, the augmented renal clearance (ARC) was an independent risk factor for prolonged antibiotic use. The author observed higher incidence of longer hospital stay, antibiotic regimen adjustments, and neurologic complications in ARC group, which was explained by the inability of renally excreted antibiotics to attain pharmacokinetic/pharmacodynamic targets.

The research on microbial resistance constitutes an integral part of our efforts to ameliorate patient care. As microbial pathogens continue to evolve and exhaust the available treatment options, the discovery of novel antibiotics, further understanding of resistance patterns and methods to overcome them should remain a priority for the scientific community. This endeavor should be a combined effort of governmental/non-governmental organizations, academic institutions and pharmaceutical industry enterprises to secure adequate funding and maximize the outcomes.

